# Trabecular Meshwork Movement Controls Distal Valves and Chambers: New Glaucoma Medical and Surgical Targets

**DOI:** 10.3390/jcm12206599

**Published:** 2023-10-18

**Authors:** Murray Johnstone, Chen Xin, Elizabeth Martin, Ruikang Wang

**Affiliations:** 1 Department of Ophthalmology, University of Washington, Seattle, WA 98195, USA; wangrk@u.washington.edu; 2 Beijing Tongren Eye Center, Beijing Institute of Ophthalmology, Beijing 100730, China; 3 Beijing Tongren Hospital, Capital Medical University, Beijing 100730, China; 4 Department of Ophthalmology, Indiana University School of Medicine, Indianapolis, IN 46202, USA; martin.elizabethann@gmail.com; 5 Department of Bioengineering, University of Washington, Seattle, WA 98195, USA

**Keywords:** aqueous valves, aqueous outflow pump, intraocular pressure, aqueous outflow, pulsatile flow, tissue biomechanics, trabecular meshwork, Schlemm’s canal valves, intraocular pressure regulation, MIGS

## Abstract

Herein, we provide evidence that human regulation of aqueous outflow is by a pump-conduit system similar to that of the lymphatics. Direct observation documents pulsatile aqueous flow into Schlemm’s canal and from the canal into collector channels, intrascleral channels, aqueous veins, and episcleral veins. Pulsatile flow in vessels requires a driving force, a chamber with mobile walls and valves. We demonstrate that the trabecular meshwork acts as a deformable, mobile wall of a chamber: Schlemm’s canal. A tight linkage between the driving force of intraocular pressure and meshwork deformation causes tissue responses in milliseconds. The link provides a sensory-motor baroreceptor-like function, providing maintenance of a homeostatic setpoint. The ocular pulse causes meshwork motion oscillations around the setpoint. We document valves entering and exiting the canal using real-time direct observation with a microscope and multiple additional modalities. Our laboratory-based high-resolution SD-OCT platform quantifies valve lumen opening and closing within milliseconds synchronously with meshwork motion; meshwork tissue stiffens, and movement slows in glaucoma tissue. Our novel PhS-OCT system measures nanometer-level motion synchronous with the ocular pulse in human subjects. Movement decreases in glaucoma patients. Our model is robust because it anchors laboratory studies to direct observation of physical reality in humans with glaucoma.

## 1. Introduction

### 1.1. A New Model of Aqueous Outflow Regulation

**Figure 1:**
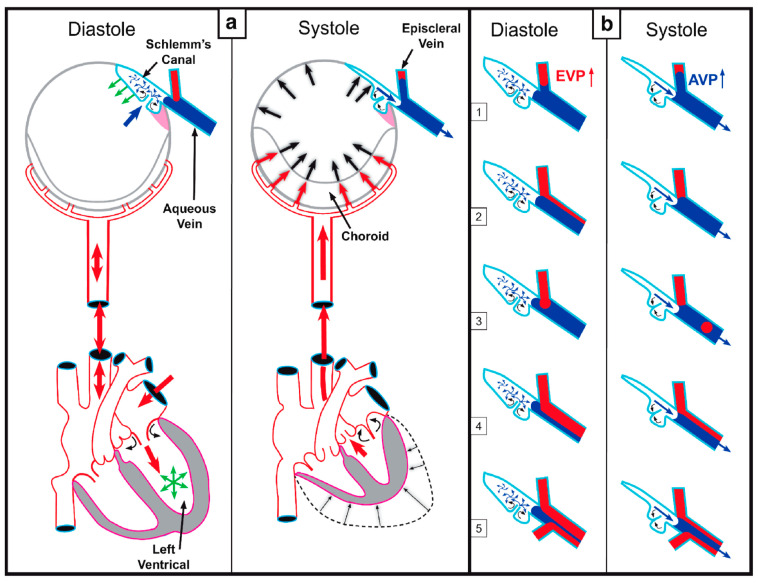
Cardiac-induced pulsatile aqueous outflow mechanisms. Cardiac source of (**a**) pulsatile aqueous outflow and (**b**) resultant pulsatile flow into the aqueous veins. The cardiac cycle results in pulsatile blood flow into the choroid, causing a rise in IOP. The IOP increase causes the TM to move outward, compressing SC. The oscillatory Schlemm’s canal (SC) compression causes a pulse wave of aqueous to enter the episcleral veins. (**b**) 1–5 represent different manifestations of pulsatile flow observed in diastole and systole. (EVP) episcleral vein pressure, (AVP) aqueous vein pressure [4].

This article reviews evidence that the aqueous outflow and vascular systems use similar physiologic mechanisms to control pressure and flow. We describe pulsatile aqueous outflow in normals that is absent in humans with glaucoma [1,2,3]. (Video 1) shows pulsatile aqueous outflow [4]. Figure 1 demonstrates pump-like behavior that controls intraocular pressure (IOP) by regulating the pulsatile stroke volume of aqueous discharge (Figure 1). (Video 2) shows stroke volume regulation of IOP) [4]. Table 1 describes the contents of videos documenting aqueous flow and motion, which are dynamic behaviors not easily captured in text and static figures.

**Figure 2:**
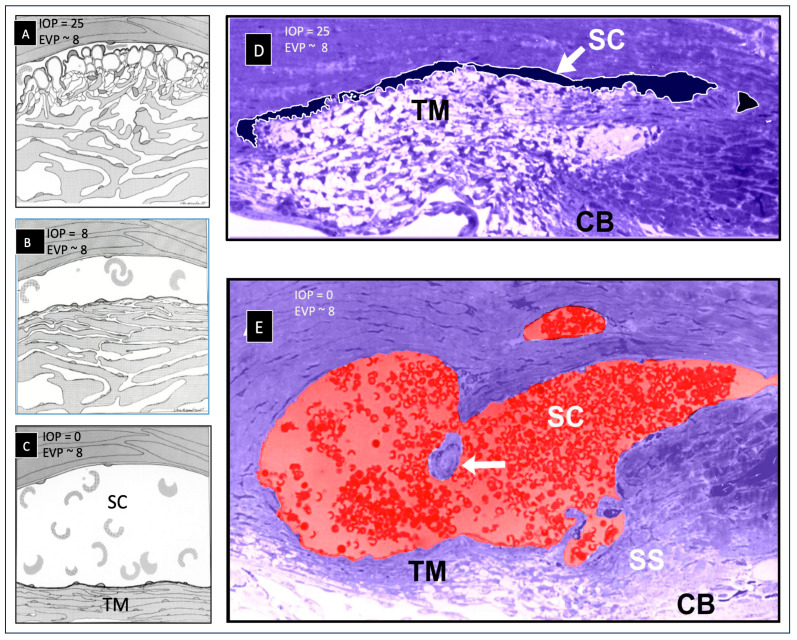
Pressure-dependent configuration of the TM, scleral spur, and ciliary body. Images (**A**–**C**) depict the tissue configuration IOP and EVP in a primate eye fixed in vivo (**D**) IOP of 25 mm Hg. (**E**) Fellow eye IOP of 0 mm Hg. (**A**) Cellular connections between the TM, juxtacanalicular cells, and SC endothelium provide tethering of the SC inner wall that prevents SC collapse. The white arrow in (**E**) indicates an SIV suspended in the canal. (TM) trabecular meshwork, (SS) scleral spur, (CB) ciliary body, (IOP) intraocular pressure, (EVP) episcleral venous pressure, (SIV) Schlemm’s canal inlet valve [4].

IOP control results from pressure-dependent tissue motion that regulates the aqueous outflow pathway dimensions that regulate stroke volume. The force of IOP determines the trabecular meshwork (TM) setpoint position between the anterior chamber and Schlemm’s canal (SC) (Figure 2).

**Figure 3:**
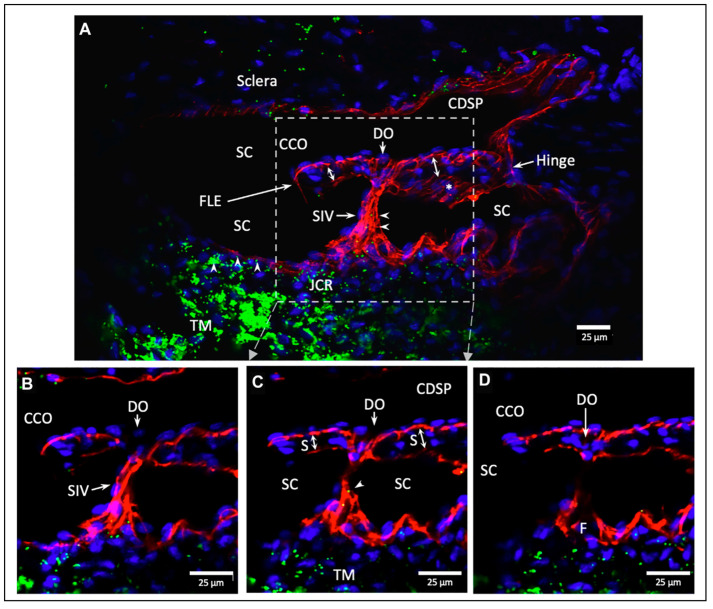
An SIV conduit connects the juxtacanalicular region to a collector channel ostia. Schlemm’s canal inner wall narrows to form an SIV (double arrows) with a continuous lumen that spans SC. The SIV passes directly through a septum to a distal opening at a collector channel ostia. The septum has an unattached edge, appearing as a flap-like extension hinging at its scleral attachment, providing mobility as a SC outlet valve. (**B**–**D**) boxed area in (**A**). Merged confocal CD31 (red), DAPI (blue), and 1 μm fluorescent microsphere (green) channels. (TM) trabecular meshwork, (F) funnel-shaped region, (SC) Schlemm’s canal, (SIV) SC inlet valve, (S) septum, (CC) collector channel, (CCO) collector channel ostia, (FLE) flap-like extension, (CDSP) circumferential deep scleral plexus, (JCR) juxtacanalicular region, (DO) distal opening (indented arrowheads); green fluorescent microspheres in the juxtacanalicular region, and the SIV lumen. See (Video 3) detailing TM/SIV/CC/CDSP relationships [6].

The ocular pulse causes the TM to undergo continuously oscillating deformation centered around the TM setpoint position (Figure 2) [5]. Section 1 TM deformation causes cyclic volume changes in SC that cause pulsatile aqueous flow from SC into the aqueous veins (Figure 3) (Section 5).

**Figure 4:**
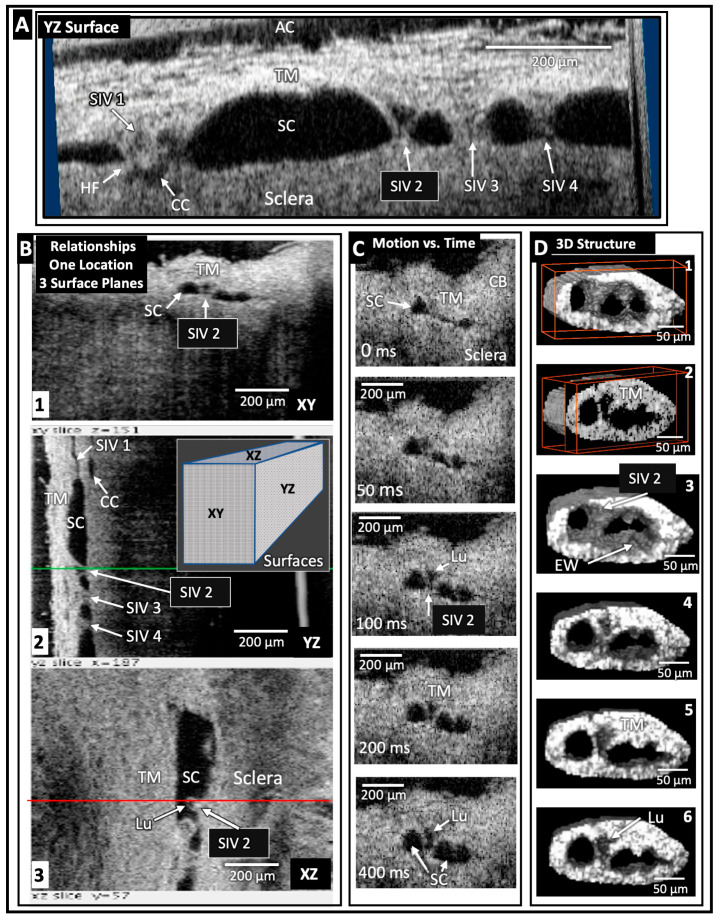
Schlemm’s canal inlet valve-like structure (SIV) 3D relationships and motion. Images (XYZ) are from 3D OCT volumes captured at 30 mm Hg SC pressure. (**A**) Four SIV crossing SC. The three surface planes in (**B1**–**B3**) clarify SIV relationships at SIV1; a hinged flap is visible at a CC entrance. (**B**) The SIV2 appearance in each plane at one location is seen in XY, YZ (green line), and XZ (red line). (**C**) Motion capture—OCT 2D imaging while SC pressure changed from 0 to 30 mm Hg. Most SIV2 lumen volume changes occurred in <200 ms. (**D**) A mask provided 3D images of SC surface appearance (**D1**) and internal structure at increased depths (**D2**–**D6**). (**C**) The funnel-shaped deformation of the TM and SIV lumen (Lu) exiting from the TM is consistent with the strain induced by pressure-dependent stresses. (SC) Schlemm’s canal, (SIVs) Schlemm’s canal inlet valves, (TM) trabecular meshwork, (EW) external wall of SC, (HF) hinged flap [12].

**Figure 5:**
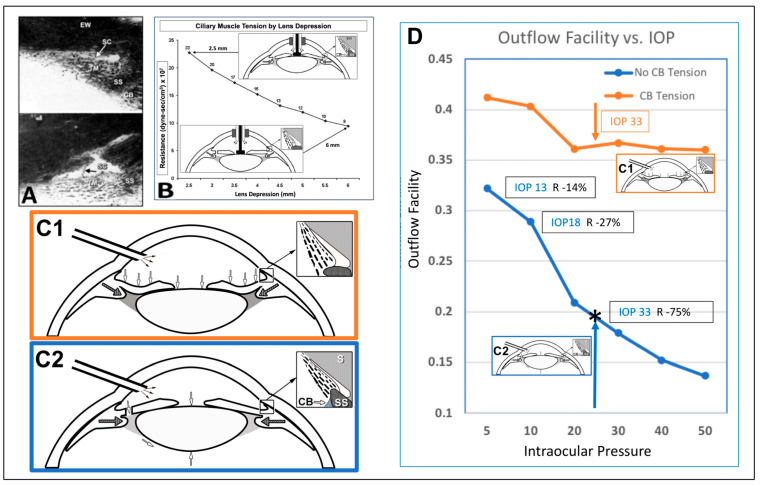
Ciliary muscle tension, intraocular pressure (IOP), and TM removal: impact on outflow facility. (**A**–**C**) depict experiments to induce ciliary muscle tension that prevents SC collapse. (**D**) illustrates the profound effects of IOP elevation on resistance and SC collapse in the absence of ciliary muscle tension. Grant’s 1958 [13] and 1963 [14] experiments were performed at an effective IOP of 33 mm Hg without ciliary muscle tension. The * indicates the effective IOP of 33 mm Hg in vivo that would result from the ex vivo IOP of 25 mm Hg pressure because of absent 8 mm Hg episcleral venous pressure, leading to the resultant high resistance. Subsequent studies performed at physiologic pressures of 13 and 18 mm Hg found that the TM accounted for 14–27% of the resistance. The addition of ciliary muscle tension to prevent SC collapse resulted in a much-improved facility that was not reduced by increasing pressure levels. (**C1**) depicts the ball valve effect of chamber deepening with resultant CB, SS, and TM tension; (**C2**) iridectomy eliminates chamber deepening and tension. (Note that 25 mm Hg IOP requires an 8 mm Hg upward adjustment for EVP correction.) (IOP) intraocular pressure, (SC) Schlemm’s canal, (EW) external wall of SC, (TM) trabecular meshwork, (SS) scleral spur, (CB) ciliary body, (EVP) episcleral venous pressure, (black arrow in (**A**)) SC inlet valve [4] Grant 1958 https://doi.org/10.1001/archopht.1958.00940080541001, Grant 1963 https://doi.org/10.1001/archopht.1963.00960040789022.

SC outlet valves (SOVs) at collector channel (CC) entrances control the flow of aqueous from SC. SIVs connect TM and SOVs [6] (Video 3). The connections ensure that the transmission of IOP-dependent TM motion is tightly linked to outlet valve opening and closing [7] (Video 4) [4]. The linkage provides the distal outflow system with a direct role in IOP control (Figure 3 and Figure 4) (Section 6 and Section 7). Evidence in humans demonstrates that the system fails in glaucoma because of abnormal TM lamellae tissue motion responses to IOP [8,9,10,11] (Figure 5) (Section 3, Section 8, Section 9 and Section 10).

### 1.2. Pressure-Dependent Configuration of the TM, Scleral Spur, and Ciliary Body

Although we use a multiplicity of laboratory techniques to validate the model, this article’s proposals are grounded in objective evidence, including direct observation of pulsatile flow throughout the outflow system in vivo in humans [1,2,3] (Video 5) [4] (Video 1) (Video 2) (Section 2). Knowledge of pulsatile flow is coupled with direct dissecting microscope observations of a mobile pressure-dependent TM [4] (Video 6). TM motion results in SC dimension changes synchronous with the opening and closing of pressure-dependent valves [7,15] (Video 7) [4].

### 1.3. Optical Coherence Tomography (OCT) Validation of Dynamic Outflow System Motion

Crucial evidence validating our model comes from optical coherence tomography (OCT) studies (Figure 4). The studies validate predictions from prior studies and introduce previously unrecognized synchrony of TM and distal system motion (Figure 4). Development of our high-resolution OCT platform permitted quantifying TM and motion at millisecond resolution in the laboratory (Figure 4). Clinical evidence and our laboratory model predicted human pulsatile outflow system motion. However, the technologies were inadequate to test the hypothesis. To solve the problem, Wang’s lab developed phase-based OCT (PhS-OCT) capable of nanometer resolution. His lab’s critical studies established pulse-dependent TM motion in humans and decreased motion in glaucoma, validating the dynamic model’s predictions [16].

We then developed a high-resolution OCT platform that permitted capturing unprecedented detail comparable to that obtained using scanning electron microscopy (SEM) [7]. Unlike SEM, our OCT system permitted imaging of fine structural details with millisecond resolution (Video 4). OCT characterized pressure-dependent relationships among the TM, structures within SC, and collector channel entrances. Prior studies of pressure-dependent behavior involved steady-state measurements in fixed tissue that prevented the resolution of time-dependent motion. Our platform permitted millisecond-level resolution of both the proximal and distal outflow structures’ movement [7].

The platform revealed synchronous motion of the TM and hinged flaps at CC entrances. The synchrony resulted from connections provided by the SIV spanning SC. Subsequent OCT studies developed an approach to characterize TM elastance/stiffness using quantitative steady-state and instantaneous TM motion studies. Glaucomatous tissue exhibited increased stiffness compared with normals and apposition and adhesion between SC walls [4]. (Video 8) shows OCT motion and stiffness in glaucoma. The OCT evidence of TM and distal valve motion led to the development of dynamic animation modeling of the unified tissue motion of the entire outflow system [7,15]. (Video 9) shows the unified motion of the entire outflow system [6].

### 1.4. The Passive Flow-Filter Model

Current textbooks and research papers regularly present a passive flow-filter model of IOP control as a definitive explanation for aqueous outflow regulation without acknowledging model limitations or rival hypotheses. The approach suggests that theory includes all relevant alternatives and that there are no viable rival hypotheses, which leads to foreclosure of further discussion. Consequently, it is difficult for research scientists and clinicians to gain awareness of the rapidly accumulating body of evidence challenging the passive model. This paper’s length reflects the need to introduce readers to unfamiliar evidence, conclusions, and the resulting concepts of a rival hypothesis.

Those engaged in clinical research and surgery may benefit from reassessing their reliance on the traditional belief system for the following reasons. (1) Minimally invasive glaucoma surgery (MIGS) documents that TM removal does not reduce IOP to episcleral venous pressure (EVP) levels as the model requires. (2) Grant’s evidence demonstrates that control of IOP is not within the TM but instead results from relationships between SC walls.

Evidence from passive flow studies points to further theory weaknesses. (1) Pores occur as a reproducible titratable artifact, and all pores may be artifactual [17,18,19,20]. No current technologies can prove or disprove their presence, leaving them in the realm of conjecture rather than theory [21]. (2) Hydraulic conductivity is orders of magnitude beyond what passive flow theory can explain, requiring a different explanation for aqueous passage into SC [18,22]. Inadequate extracellular matrix is present in the juxtacanalicular region to explain resistance, suggesting that the passive flow-filter model’s fundamental thesis is unsupported [23,24,25].

The passive flow-filters theory’s dependence then rests on an ad hoc funneling idea proffered to sustain the model’s framework. However, the concept was not formulated based on empirical data demonstrating funneling behavior and remains unsupported by meaningful objective evidence. No current technology can prove or falsify funneling behavior, leaving funneling in the realm of speculation and best described as a conjecture rather than a theory [21]. Review articles by authorities [23,24,25] point out that funneling conjecture is now the sole means of retaining support for the passive flow theory of resistance control.

## 2. Noninvasive Aqueous Angiography: Stealth Paradigm Transformation

Slitlamp development introduced a technology that permitted the most crucial breakthrough discovery of the 20th century: direct observation of aqueous flow from SC to the episcleral veins. Our current concepts of aqueous outflow and its abnormality in glaucoma rely on Ascher’s discovery of the aqueous veins [1,2,3]. Scientists actively disputed the idea of aqueous circulation before his discovery. By studying readily available videos, we can easily verify pulsatile flow in the aqueous veins. For example, see the aqueous vein pulsations in (Video 1) and (Video 2).

Evidence was insufficient to support the theory of aqueous circulation before Ascher’s discovery. The concept of angle-closure glaucoma was disputed; open-angle glaucoma was an enigma. Reports by Asher and others removed the controversies, opening the way for the widespread use of peripheral iridectomy, and identifying circulation and its blockage at the level of the outflow system [1,2,3] provided the rationale for outflow drugs and MIGS surgery, which are the subjects of this report.

Pulsatile flow has been recognized as a salient feature since the aqueous veins were first identified. DeVries noninvasively explored the undisturbed distribution of aqueous veins. Aqueous outflow in humans is highly asymmetric and concentrated in the inferior hemisphere (87%), particularly the inferior nasal quadrant (56%), a distribution well documented in his exhaustive report [26].

Aqueous vein patterns are likely stable for a lifetime [3], with two or three typically visible, four and, at most, five are present only occasionally [3,26]. Stepanik’s videography measured the velocity and volume of pulsatile flow in the aqueous veins under noninvasive, physiologic conditions. His quantitative study demonstrated that two aqueous veins could carry all aqueous outflow [27,28].

### 2.1. Pulsatile Flow into Aqueous Veins Requires Specific Structural Outflow System Features

The arterial, venous, and lymphatic systems all have continuous, pulsatile flow as components of vascular circulatory loops. The aqueous outflow system is one such circulation. An arterial arm of the loop delivers aqueous from the heart to the eye, then returns it to the venous system and the heart. Pulsatile mechanisms in the systemic veins and lymphatic vessels return fluid to the heart [29]. Pulsatile flow requirements for the vasculature are three-fold: (1) compressible chambers, (2) valves, and (3) an energy source. Sections between venous and lymphatic system valves act as mini ventricles or chambers [29].

Initial lymphatics and small veins rely on energy from the cardiac system to alter tissue turgor, causing chamber compression during systole and relaxation during diastole. Valves prevent backflow. Pulsatile flow in the outflow system imposes equivalent requirements to permit pulsatile flow. Requirements are compressible chambers, valves, and a chamber wall that can respond to the cardiac pulse. As noted in the following sections, pulsatile aqueous flow is well-characterized in humans [1,2,3]. Structural features and functional behavior within the outflow system also explain and predict pulsatile flow behavior [7].

### 2.2. Pulsatile Flow in Aqueous Veins, Intrascleral Channels, Collector Channels, and SC

In response to cardiac-dependent changes in choroidal volume, the anterior chamber experiences continuous pulsatile alterations in pressure [30]. Pressure oscillations force the TM outward in systole, followed by recoil in diastole. SC volume oscillates as a result.

SC inlet valve-like structures (SIVs) are regularly distributed around the circumference of SC in human eyes. The SIVs have a funnel-like proximal origin from SC inner wall endothelium, and their lumen communicates with the juxtacanalicular space. The SIVs develop a cylindrical shape and attach to SC’s external wall at CC entrances [6].

In response to the ocular pulse, waves of aqueous propagate from funnel-shaped origins at SC’s inner wall into a cylindrical region, culminating in aqueous ejection from the distal end. The ejected aqueous creates swirling eddies in SC as it mixes with blood. A recurring pattern over thirty cardiac cycles demonstrates that aqueous entry to SC is synchronous with the ocular pulse [5]. Objective evidence using direct observation is visible in readily available peer-reviewed video material [4], for example, (Video 5) shows pulsatile aqueous flow into SC (start the video at 2:19 min to see SC pulsatile flow).

A generalizable pattern is apparent in the propagating wave of clear aqueous. A specific configuration outlines funnel-shaped and cylindrical areas in each cycle as aqueous moves through a clearly defined constraining structure. The shape configuration changes rapidly as aqueous passes through the conduits, demonstrating that their constraining walls are highly compliant. Real-time pulsatile aqueous entry into SC in humans provides a benchmark for assessing the validity of the dynamic model. Blood-stained aqueous moving outward in CC and intrascleral channels in synchrony with the cardiac pulse provides confirmatory evidence of pulsatile flow in the outflow system [5]. To summarize, pulse waves of aqueous progress through the TM into SC and then into CC, intrascleral channels, aqueous veins, and, finally, episcleral veins.

### 2.3. IOP Oscillations and Pressure Transients

The AC pressure and volume continually oscillate due to cardiac-dependent changes in choroidal volume. Eye movements and blinking cause the IOP to undergo multiple transients above baseline each minute. In healthy people, dynamic contour tonometry demonstrates an ocular pulse amplitude of 3 mm Hg ranging from 0.0 to 7.2 mm Hg [31].

Blinking and eye movements cause recurring instantaneous IOP spikes reaching 20–30 mm Hg. Eye movements are frequent in daily activities, with approximately 170,000 daily saccades. Monkey eye telemetry demonstrates that the eye experiences transient IOP spikes 2.5 mm Hg above baseline about 5000 times per hour [32]. These oscillatory and transient pressure spikes are the source of pulsatile aqueous outflow from SC.

### 2.4. Clinical Studies Confirm Rapid Trabecular Meshwork Motion in Humans

TM motion effects can be directly observed by watching rapid blood reflux into SC [33,34,35]. Episcleral vein compression with the flange of a goniolens causes a reversal of pressure gradients with EVP > IOP. The TM moves toward the AC within seconds as blood refluxes into SC in response to the pressure gradient reversal. Pressure gradient reversal in living primate eyes followed by histology studies confirms clinical findings [36]. In vivo studies demonstrate that blood widely dilates SC. SC dilation results from the complete collapse of the inner portion of the TM tissues with associated outward movement of all the trabecular lamellae. The directly observable blood reflux and SC dilation in humans can be coupled with comparable evidence in living primates that experience pressure reversal. In each case, the TM rapidly deforms in response to the pressure changes. TM motion associated with SC filling slows and eventually stops as glaucoma progresses. The finding is well documented in multiple quantitative clinical studies [33,34,35,37,38].

## 3. Outflow Abnormalities in Glaucoma—Directly Verifiable Clinical Evidence

Initial aqueous vein studies identified pulsatile flow as a salient feature [1,2,3]. Soon after the initial studies, Ascher and others published evidence that pulsatile flow slows and eventually stops as glaucoma worsens [3].

### 3.1. The Aqueous Influx and Compensation Maximum Outflow Tests for Glaucoma

Aqueous vein pulsatile flow increases in normal subjects when EVP rises. We can clinically observe the pulsatile flow increase when pressure on a distal episcleral vein causes pressure in the more proximal vein to rise. An increased stroke volume per pulse causes aqueous influx into adjacent previously blood-filled ESVs. The pulsatile aqueous influx reflects the TM’s ability to alter its stiffness/elastance in response to changing the pressure differential across its surface. A more vigorous distention and recoil then occur with each ocular pulse wave. In glaucoma, the outflow system loses the ability to increase pulsatile flow in response to increasing EVP [1,3], a behavior consistent with the loss of elastance of the TM.

When IOP increases in a compensation maximum test, the stroke volume of pulsatile aqueous flow also increases in normal subjects. In contrast, pulsatile flow decreases and eventually stops when IOP increases in glaucoma patients [39,40]. Clinicians use an ophthalmodynamometer that presses on the eye to increase IOP. The instrument provides a quantitative assessment of the induced pressure. The test assesses the ability of the TM to withstand increased IOP distending forces above its homeostatic setpoint. The TM loses its ability to withstand distending forces in glaucoma, resulting in an initial decrease and an eventual absence of pulsatile aqueous outflow as glaucoma worsens [39,40].

### 3.2. Miotic Responses Identify a Mechanistic Cause of the IOP Increase in Glaucoma

Pulsatile aqueous outflow slows and then stops in advanced glaucoma. Miotics reduce IOP in glaucoma patients, a response preceded by increased pulsatile aqueous outflow. As the duration of mitotic action ends, pulsatile flow stops. Recurrent elevation of IOP follows [1,3]. Ciliary muscle contractile responses increase in response to miotics [41]. Contractile responses of the muscle cause increased tension on the scleral spur and TM lamellae. The TM tissues move away from the SC external wall, and the TM lamellae experience increased tension-induced stress (Figure 5). The increase in TM lamellae stress alters their elastance, restoring their ability to distend and recoil within their homeostatic response range [5].

The ciliary body’s role in controlling aqueous outflow has robust experimental support (Section 8). In the absence of ciliary muscle tone, increasing IOP results in IOP elevation and SC collapse in ex vivo eyes. The introduction of ciliary body tension allows the tissue to respond with stable resistance over an extensive range of pressures [9]. Ciliary muscle tension and outflow resistance are tightly linked. Laboratory studies support the conclusion that ciliary muscle tension is a crucial parameter of aqueous outflow regulation in both normal and glaucoma eyes [8,9,10,11,41,42].

## 4. TM—Mobile Wall of a Compressible Chamber Called SC

### 4.1. TM Structural Organization and Composition That Determines Function

Trabecular lamellae typically originate at Schwalbe’s line, quickly branching into three lamellae. The lamellae course posteriorly, and then further branch to form a series of 8–14 parallel beams or sheets, each about 5–12 µm in thickness (Figure 2). The branching results in a fan-shaped appearance of the TM with the base of the fan at Schwalbe’s line (Figure 2). The trabecular sheets are generally circumferentially arranged, with the sheets parallel to one another and the limbal circumference [43].

The trabecular lamellae of the corneoscleral TM extend from Schwalbe’s line posteriorly in a meridional fashion. Their posterior anchoring distributes about equally between the scleral spur and the ciliary muscle tendons. Lamellae near the AC are considerably thicker than the ones close to SC (Figure 3). The composition of the corneoscleral trabecular lamellae is similar to that of other organ systems with marked elasticity and compliance, such as the lung, blood vessel walls, and tendons.

### 4.2. TM Composition Determines TM Lamellae Energy Storage and Release

The trabecular lamellae’s organization and distribution of elastin and collagen are like that of a tendon. Pulsatile changes in IOP are forces that cause oscillatory TM tissue loading. The composition can explain the observed reversible TM deformation. TM lamellae endothelial cells have a rich endowment of cytoplasmic organelles responsible for protein synthesis and secretion. TM lamellae experience about 30 million oscillations per year. Constantly optimizing elastin and collagen fiber properties is essential for maintaining homeostasis in such a dynamic environment. Cytoskeletal elements in the trabecular endothelial cells and their cytoplasmic processes are microfilaments (F-actin), intermediate filaments (vimentin), and microtubules (alpha-tubulin) [43].

### 4.3. TM Lamellae, Juxtacanalicular Cells, and SC Inner Wall Connect by Cell Processes

Trabecular lamellae endothelial cells project many cytoplasmic processes into the intertrabecular spaces. Processes of adjacent lamellae join, creating cellular connections. TM lamellae cytoplasmic processes from the TM lamellae also project into the juxtacanalicular space, attaching to juxtacanalicular cell processes. The relationships result in the TM lamellae being tethered to the SC inner wall endothelium. The TM lamellae move outward as IOP increases because the numerous processes tether them to the JCT and SC endothelium [20,36,44,45,46,47] (Figure 2). The inner wall endothelium initially moves outward, but the TM lamellae process connections limit the SC inner wall distention into the SC lumen.

### 4.4. Immediate Responses to Pressure Changes Result from Continuous Tensile Forces

SC endothelium experiences continuous pressure-dependent baseline, oscillatory, and transient stresses at physiologic pressures, as illustrated using in vivo fixation studies [20,36,44,45,46]. The tensile stresses at SC inner wall endothelium transmit to the TM lamellae through the numerous tethering cytoplasmic processes. The force of IOP exerts continuous uniformly distributed tensile forces on SC endothelial cells, juxtacanalicular cells, and endothelial cells covering the trabecular lamellae.

### 4.5. Tensile Stress Status and Changes—An Information-Dense Sensory System

The IOP-induced force creates tension on SC’s inner wall endothelium. The stresses are transmitted to the entire TM complex through cellular process connections. The uniformly distributed tension provides TM cells with information that permits instantaneous, simultaneous sensing of pressure and changes in pressure. The pressure-dependent sensory input enables TM cells to maintain optimized elastance through mechanotransduction mechanisms. Cardiovascular system walls elsewhere experience comparable pressure-dependent sensory input and responses [29].

TM biomechanical properties determine SC dimensions. Control of TM biomechanical properties determines how far the TM distends into the canal. In unfixed ex vivo eyes, experimentally induced pulsatile motion is directly observable. Pulse-dependent TM distension causes SC progressive collapse. After the pulse wave stops, the TM recoils to the baseline configuration. Direct observation of the pulsatile TM behavior is visible using videography. Scale bars on the images permit the capture and quantitation of TM motion speed and amplitude in this living tissue [4], for example, see (Video 6) of TM motion and SC collapse.

### 4.6. New OCT Technology Permits TM Elastance Determination

TM biomechanics quantitative studies have been significantly advanced by recently developed high-resolution spectral domain OCT (SD-OCT) capture of movement in ex vivo TM tissues. Using these technologies, we explored a new TM biomechanical parameter, elastance. Elastance is a measure that quantifies the ability of a tissue surrounding a volume to store and release energy [48]. The TM is the deforming tissue that determines the volume of SC. In this setting, the TM is the tissue storing and releasing energy as it responds to IOP changes by deforming. Stiffness is an alternative term for elastance. Our studies permitted us to generate TM elastance curves. The curves characterize the TM tissues’ stiffness and ability to respond to pressure changes. They explore both the dynamic volume changes and response speeds. TM distention and recoil are very rapid, occurring within about 250 milliseconds [7,12].

Another technique, PhS-OCT, provides motion sensitivity at the nanometer level with a 1000 nm dynamic range [7,48]. Recent ex vivo experimental platforms and algorithm developments demonstrated measurement feasibility. Subsequently, PhS-OCT was translated into a clinical imaging system that identifies TM pulsatile flow in vivo in humans and motion changes in glaucoma. Diurnal IOP fluctuation is an elusive but essential parameter in glaucoma management. PhS-OCT can assist in assessing IOP control by identifying the propensity for diurnal changes [16,47].

## 5. SC Inlet Valves (SIVs): Discovery and Operating Room Documentation

SIVs were discovered during experiments that identified SC as a dynamically compressible chamber [36] (Figure 2). A study on the SIVs or informed commentary about their presence, absence, or behavior is not feasible without the dilation of SC. The SIV course is circumferentially in SC. At normal pressures, the distended TM tissues obscure the presence of SIVs, their origins at the level of the TM, and their insertion at CC entrances along the SC external wall.

Initial awareness that the outflow system could function as a pump resulted from three discoveries: (1) pressure-dependent TM motion permits SC to behave as a compressible chamber, (2) an SIV has a pressure-dependent lumen, and (3) an SIV acts as a conduit that allows aqueous passage directly from the juxtacanalicular space into SC.

### 5.1. Multiple Imaging Modalities Confirm SIV Presence and Function

Operating room unroofing of SC provides directly observable evidence of SIV structural features, function as conduits carrying aqueous, and tensile nature of the connections with the inner and outer wall of SC [5]. (Video 5): surgical stretching and disruption of SIV by Stegmann (starting at 1:10) provides evidence. The SIVs undergo structural failure in response to experimentally induced axial stresses, leading to rupture and aqueous gushing from the disrupted lumen [5]. Direct observation of aqueous flow from the AC through the SIV to SC is made possible using operating room gonioscopy (see (Video 5) for evidence of pulsatile aqueous flow into SC starting at 2:19).

### 5.2. SIV Structure and Motion Responses: Clinical and Laboratory Correlation

Many modalities demonstrate the SIV structure, the effects of ciliary muscle tension, and pulse-induced motion. They also show that pressure-dependent TM motion creates stress on the SIV that induces movement of the SC outlet valves (SOVs). Modalities that characterize the SIV structure are the dissecting and brightfield, phase contrast, differential interference contrast [4], and confocal microscope [6]. Additional modalities that characterize features and relationships include standard and blockface scanning electron microscopy (SEM) [5,36,49] and spectral domain OCT (SD-OCT) [7,12,49].

### 5.3. Protocols to Study SIV

Protocols to assess SIV motion include in vivo fixation at controlled steady-state pressures [5,15,36], controlled ciliary muscle tension, and direct pulsatile infusions at the dissecting microscope [4]. In vivo imaging protocols include high-resolution OCT while perfusing at steady-state pressures and during experimentally induced pulsatile motion. The findings reveal axial and radial SIV lumen dimension changes [4]. (Video 4) of linked TM/CC motion PhS-OCT demonstrates that IOP-dependent TM motion changes must induce SIV configuration changes because the SIVs connect to both the TM and SC external wall (Section 4.3).

**Figure 6:**
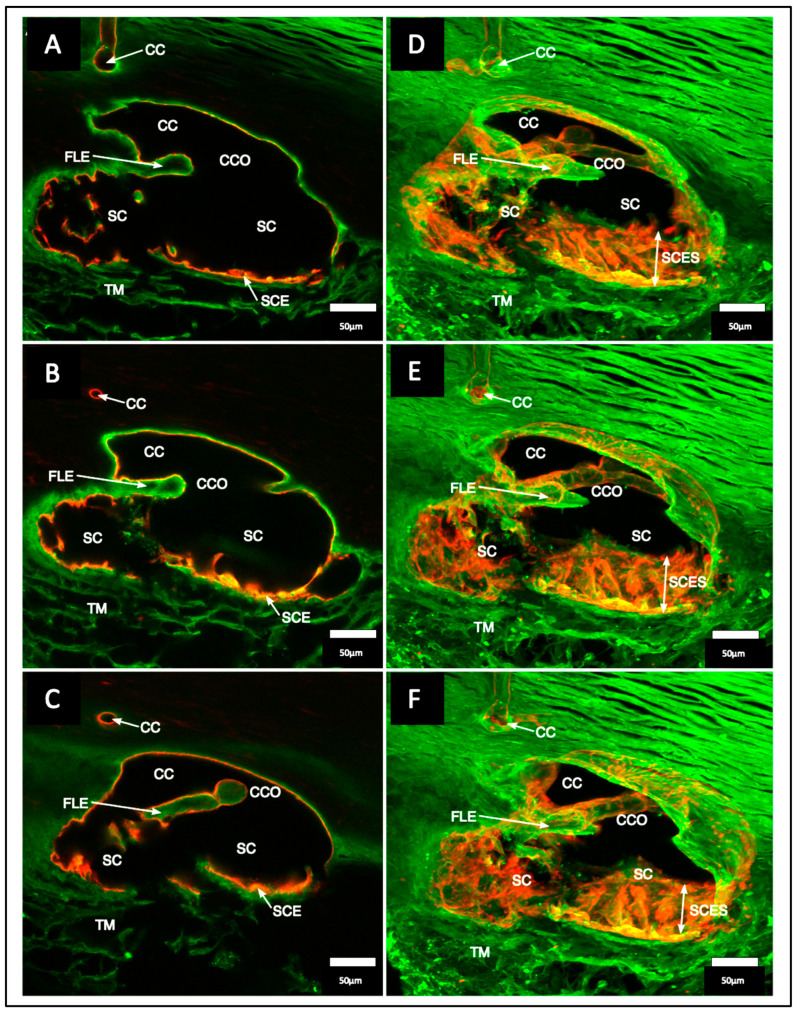
Confocal stack projections display Schlemm’s canal flap-like collagenous extensions at collector channels. (**A**–**C**) are 2D and (**D**–**F**) are 3D projections of a 129 μm stack of merged CD31 (red) and Col type 1 (green) images. Viscodilation of Schlemm’s canal (SC), clarification, and deep stack projections characterize the organization and relationships between tissues surrounding collector channels (CCs) and their ostia (CCO). 2D images show Schlemm’s canal endothelium (SCE) and Schlemm’s canal inner wall endothelial surface (SCES) is visible in the 3D stack projection. Flap-like collagenous extensions (FLEs) from the sclera wall protrude into SC. The unanchored distal end of the FLE provides a hinged configuration. (TM) trabecular meshwork [6].

**Figure 7:**
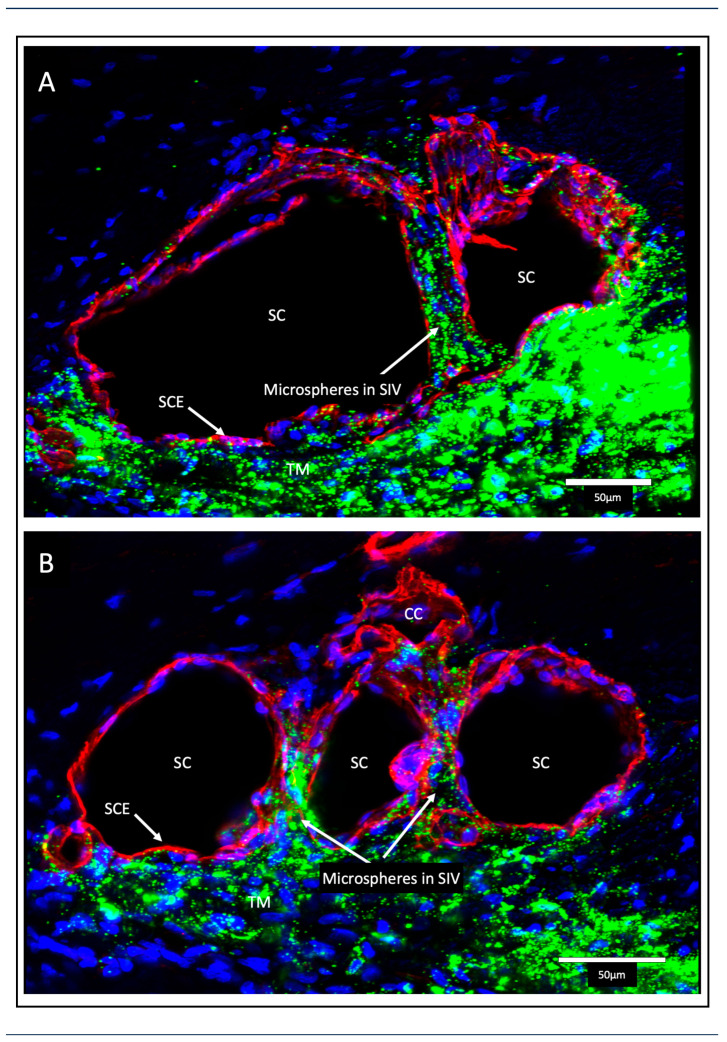
Schlemm’s canal inner wall valve-like structures. Tracer studies (**A**,**B**) merged CD31 (red), DAPI (blue), and 500 nm fluorescent microsphere (green) channels. The stack size is 40 μm in (**A**) and 43 μm in (**B**). Schlemm’s canal (SC) inner wall endothelium forms a valve-like structure (SIV). The SIVs arise from the SC inner wall endothelium (SCE) that forms the outer wall of the trabecular meshwork (TM). The funnel-shaped configuration extends into SC to form a cylindrical conduit attaching to the SC external wall at collector channels (CCs). The TM fills with fluorescent microspheres. The microspheres also fill the juxtacanalicular region, the SIV, and their connections with intrascleral channels (ISCs) [6].

The SIV endothelial walls and the lumen can be traced from their funnel-shaped origin at the SC inner wall to their outer wall attachments. The technique involves SC lumen viscoelastic dilation, clarification, IHC labeling, tracers, and confocal microscopy. These combined approaches establish the constituent properties of the walls and lumen of the SC inlet valves [6] (Figure 3Figure 6 and Figure 7). Labeling studies reveal that SIV walls are those of a vascular endothelium continuous with SC inner wall cells. Microspheres perfused into the AC can be traced as they pass from the juxtacanalicular space of the TM into the SIV lumen, followed by passage into CC entrances and circumferential vascular channels (CVCs) [6].

Perfusion of nucleated avian red cells into the AC results in their passage through the entire length of the SIV lumen, as revealed with light and TEM [5]. In vivo reduction of IOP below EVP results in blood filling the SC lumen. At the same time, primate red cells pass through the entire SIV length, reaching the JCS level. No red blood cells or plasma pass directly across the distended endothelium of the SC inner wall [5,15].

## 6. Schlemm’s Canal Outlet Valve (SOV) Structure and Function

### 6.1. Circumferential Channels Parallel to SC in Humans: The Definitive Study

Ramirez et al. deserve credit for the discovery, clear articulation of anatomy, and embryogenesis of what they describe as “an outer collector that runs parallel to SC” [50]. Our later reports, unaware of the Ramirez group’s discovery, used the terms circumferential deep scleral plexus, circumferential vascular channel, and intrascleral collector channel for the “outer collectors”. The group’s landmark discovery compares with that of Ashton’s vascular casting studies [51], which established pathways from SC to the episcleral vessels. The “outer collector” clinical importance is comparable to that of the TM in understanding pressure control mechanisms and identifying surgical treatment targets.

The Ramirez group studied the embryology of distal “outer collector” pathways in 60 eyes. They explored five gestation intervals beginning at 24 weeks and compared them with the appearance after birth at two months, eight years, and adulthood. At 24 weeks, the anlage of “outer collectors” was present; at 36 weeks, it was clearly defined, and at two months after birth, its circumferential connections continued to develop. At eight years, the structures compared with those of adult. The structures were circumferential extensions of collector channels that arose from the SC ectomesenchyma. The episcleral plexus developed separately.

In seven randomly selected adult eyes, the mean number of CC connecting to the “outer collectors” was 39 (±6.24). Circumferential “outer collectors” were divided into sectors but were continuous for as much as 120°. Numerous ramifications from the external wall of the “outer collectors” formed intrascleral channels entering the episcleral veins. The report’s description of the circumferential channels appears identical to that in our group’s studies. Our later independent studies were performed without awareness of the Ramirez detailed report. However, our studies confirmed the earlier work using microscopy by manipulating fresh tissue, light microscopy, SEM, micro-vascular casting, and high-resolution OCT.

### 6.2. SOV Structural Features Assessed Using Multiple Approaches

IOP regulation includes distal resistance regulation as an essential factor. However, structural relationships and behavior that can control distal resistance are unclear [52]. Below, we describe evidence of distal resistance-controlling structures and their functional behavior. Studies include capturing the effects of direct manipulation while imaging with a microscope [4], histology [5,49], SC viscoelastic dilation, and examination of three thousand individual SEM preparations involving images at 12,000 locations and magnifications [7,49]. Studies also include microvascular casting [4,49] (Figure 5), tissue clarification [6], confocal microscopy [6], and IHC [6].

### 6.3. Septa, Hinged Flaps at CCs, and a Second Compressible Chamber

Septa oriented parallel to the external wall of SC separated SC from a second compressible chamber (Figure 6). Regularly recurring septa are found along the external wall of SC. At CC entrances, they appear as hinged flaps (Figure 3 and Figure 6). A circumferentially oriented deep scleral plexus of channels (CDSP) extends around much of the outer wall periphery of SC, and the lumen communicates directly with CCs, SIVs, and the juxtacanalicular region (Figure 7).

The CDSP are partitioned from SC by thin septa and are also referred to as circumferential vascular channels (CVCs) [4,5,6,7,12,49]. Septa are thin and highly mobile. The properties of the septa result in high mobility, permitting pressure-dependent compression of the CDSP/CVC.

**Figure 8:**
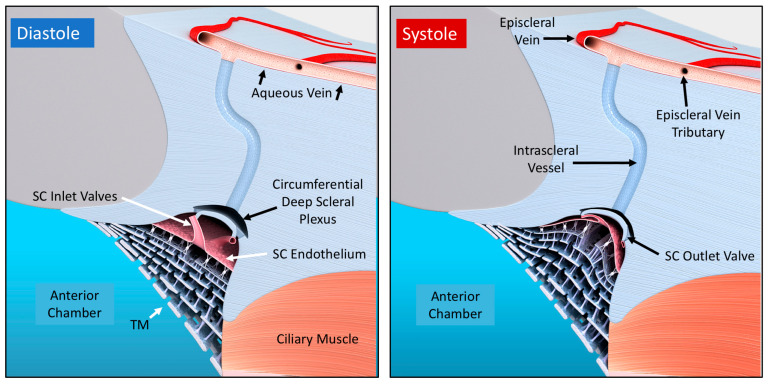
Aqueous pump model incorporating valves and compressible chambers. From the resting state in diastole, systole induces an intraocular pressure (IOP) rise, causing an ocular pulse wave in the anterior chamber (AC). The trabecular meshwork (TM) distends as IOP rises. TM distention causes Schlemm’s canal (SC) to narrow, reducing its volume while forcing fluid through the collector channel (CC) outlet valve entrances which are initially in an open configuration, followed by septa movement outward, forcing aqueous out of the CDSP. TM recoil during the following diastole causing CC entrances to open into the circumferentially oriented deep scleral plexus (CDSP). Aqueous flows into the intertrabecular spaces, into SC through the conduits of the SC inlet valves, and into CDSP. The cycle then repeats. The proposed anatomic relationships and pressure-dependent sequences are provisional and warrant further study [4].

The septa mobility results in the CDSP acting as a second compressible chamber, distal but adjacent to SC (Figure 8). CC entrances are typically perpendicular to the circumference of SC and course only a short distance before entering the CVC. When sections are cut in an oblique plane, SEM captures the entirety of the relationships, including a short CC entrance, circumferential intrascleral path of the CVC, and radial intrascleral channels exiting the CVC that pass to the episcleral veins. Precise positioning is required because the relationships disappear with the slightest movement in any XYZ plane or rotation around the XYZ axis.

Only recently has the consistent presence and likely functional significance of a second compressible chamber adjacent to SC come into focus. A study identified and quantified relationships with SEM using limbal segments from human and primate eyes. About half the segments had CVC that coursed parallel to SC. In the segments, the mean length of SC was 1130 ± 586 µm, and CDSP/CVC was 565 ± 486 µm. The CDSP/CVC length is also a measure of the septa length that separates the two chambers. The mean scleral septa diameter was 23 ± 11 µm, and the thin diameter relative to length explained the septa mobility. SD-OCT and confocal microscopy 3D volume analysis using serial imaging, 3D reconstructions, associated projections, and SD-OCT further confirmed CDSP/CVC characteristics [6,12].

### 6.4. SOV Structural Features Assessed Using Microvascular Casting Approaches

**Figure 9:**
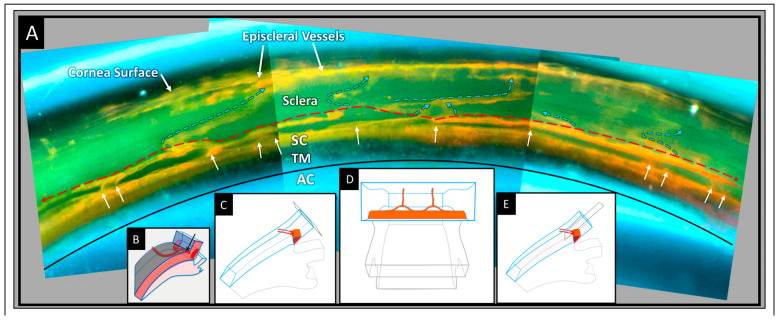
Circumferentially oriented deep scleral plexus visualization. A microvascular cast of the outflow system following tissue clarification. (**A**) The transparent TM is between the black curved line and SC. Collector channels arise from the outer wall of SC (white arrows) and connect to a circumferentially oriented deep scleral plexus (CDSP) (thin dashed red line). The CDSP forms a relatively continuous communicating ring adjacent and parallel to SC. Intrascleral vessels exit the CDSP and pass through the sclera (blue arrows) to the eye’s surface, where episcleral and aqueous veins are visible. Between SC and CDSP are long, thin, collagenous septa, which are transparent and seen as a void between SC and the CDSP. The CDSP provides a compressible valve-like conduit/chamber because it opens and closes with pressure-dependent septa movement. (**B**–**E**) illustrate the need to visualize the CC/CDSP connections through the cornea surface to capture their uniform perpendicular plane of exit from SC. (AC) anterior chamber, (TM) trabecular meshwork, (SC) Schlemm’s canal [4].

The technique injects microvascular casting material into SC. Tissue clarification follows, allowing imaging deep into the tissue, which is ideal for exploring distal scleral relationships (Figure 9). A clarification technique, new to ophthalmology, uses benzyl alcohol/benzyl benzoate (BABB) that rapidly clears the scleral tissues in the distal outflow pathway (Figure 7) [6].

We found that cutting specimens at the corneoscleral interface was necessary to define the CC exit sites clearly. Visualization through the corneoscleral interface permitted us to visualize the CC perpendicular to where they exit the SC lumen (Figure 9). The protocol enables grasping global relationships involving SC, CC, septa, and CDSP/CVC. The ensemble provides the structural framework for understanding how SOV can function as outlet valves [4,6,49].

### 6.5. SOV Structure and Related Motion Motion—OCT Imaging Approaches

High-resolution SD-OCT imaging permits tracing TM, SC, SIV, CC, septa, and CDSP/CVC configuration changes. The experimental platform enables examination under steady-state conditions at different pressures. Experimentally controlled changing of pressure gradients while imaging permits quantifying motion in real-time with millisecond resolution in NH primates and human eyes [7,12]. The related new technology (PhS-OCT) provides millisecond motion resolution in glaucoma patients and identifies motion differences compared with normals [16,48].

The discovery of a collapsible chamber distal to SC was unexpected, and the evidence was initially discounted. However, regular organization, occurrence, and behavior are consistent with an essential role in controlling IOP. Multiple segments that all function to control flow, like miniventricles in series, are a feature of the lymphatics. The complex circular torus of SC may have used a similar evolutionary solution using an in-parallel approach adapted to the peculiar needs of a torus.

## 7. Outflow Regulation by an Aqueous Pump

### 7.1. First Mechanism: A Baroreceptor—IOP-induced Stretch—A Sensory System

**Figure 10:**
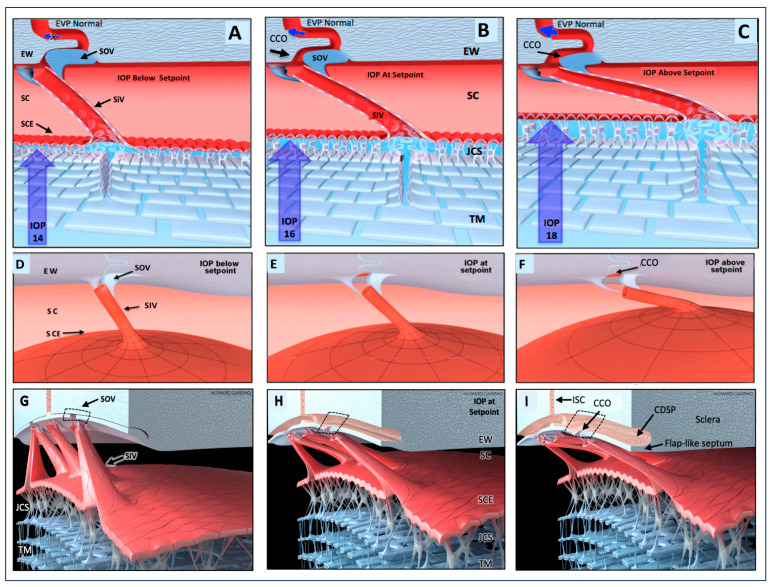
Depiction of the pressure-dependent relationships between trabecular meshwork, Schlemm’s canal, and valve-like structures. Images (**A**,**D**,**G**) depict the outflow system configuration at an intraocular pressure (IOP) below a homeostatic setpoint, images (**B**,**E**,**H**) are at the setpoint, and images (**C**,**F**,**I**) are above the setpoint. In this provisional model, aqueous passes through the trabecular meshwork to the juxtacanalicular region and then flows through Schlemm’s canal inlet valves into the SC lumen at collector channel ostia present at the external wall of SC. An SC outlet valve consists of a mobile flap-like septum between SC and a circumferentially oriented deep intrascleral vascular plexus. The circumferential plexus serves as a closable conduit or chamber controlling aqueous flow. Blue arrows with increasing size indicate increased flow. See (Video 9) for animation of dynamic tissue motion and aqueous flow sequence. (TM) trabecular meshwork, (SC) Schlemm’s canal, (SCE) Schlemm’s canal inner wall endothelium, (SIV) SC inlet valve, (JCS) juxtacanalicular space, (EW) SC external wall, (CDSP) deep intrascleral vascular plexus, (IOP) intraocular pressure, (SOV) SC outlet valve, (CCO) channel entrances ostia, (ISC) intrascleral channel. See (Video 2) showing outflow feedback relationships that can control IOP [6].

The outflow system controls the return of aqueous to the heart. As a vascular circulatory loop, the outflow system functions by four homeostatic mechanisms identical to other vessel loops [29] (Figure 10). First, the TM is tensionally integrated and prestressed by IOP under physiologic conditions, as demonstrated with in vivo fixation (Figure 3) [15,20,36,44,46,53,54,55]. Continuous IOP-induced prestress links the TM configuration and IOP. Because of the prestress, the TM responds to small changes in IOP with profound shape changes. Optimized elastance causes TM deformation to track IOP with high fidelity. The close coupling of configuration and pressure permits the TM to function like other systemic baroreceptors, such as those of the aortic arch that sense stress-induced tissue deformation resulting from changes in volume that are tightly linked to pressure [29].

### 7.2. Second Mechanism: SIV Link IOP-Induced TM Motion to SC Outlet Valves

Coupling the TM to SOV through SIV connections provides a second regulatory mechanism (Figure 3, Figure 4, Figure 7 and Figure 8). The SIV connections between the TM and SOV hinged flaps at CCs are obliquely arranged [7]. Increasing pressure-induced outward movement of the TM results when IOP increases and permits fine adjustments of SOV dimensions through tensional integration and continuous IOP-induced prestress.

An increase in SOV lumen size can increase aqueous flow and reduce IOP. When IOP decreases, the TM moves toward the AC, permitting restoration of the setpoint. TM inward movement reduces tension on the obliquely arranged SIV. The reduced tension results in reduced flow IOP return to the homeostatic setpoint. A conduit-like behavior can be achieved close to the setpoint with the valves remaining slightly open. At the setpoint, visible pulsatile flow in the aqueous veins may be minimal or absent. When IOP rises significantly above the setpoint, the pulsatile flow becomes manifest [40]. See Video 2 showing stroke volume regulation of IOP.

### 7.3. Third Mechanism: Stroke Volume Regulation As an Innate Physical Feedback Loop

A third IOP regulatory mechanism is the control of stroke volume. The ocular pulse increases as IOP rises, thus increasing TM oscillatory amplitude. The increased TM amplitude increases SC volume, resulting in increased pulsatile aqueous discharge from SC to the aqueous veins [4]. See (Video 2) showing stroke volume regulation of IOP. 

The pressure-dependent TM motion responses provide a physical, sensory error detection mechanism to identify pressure elevation. The physical TM motor responses act as a feedback mechanism. An increase in IOP causes increased stroke volume, restoring pressure to its homeostatic setpoint. When IOP is below the setpoint, TM cyclic decreases, the per pulse stroke volume decreases, total aqueous flow decreases, and IOP rises, returning to its homeostatic setpoint [4].

### 7.4. Fourth Mechanism: Pulsewave-Dependent Shear Stress in TM and Distal Pathways

In 2004, Johnstone first recognized evidence leading to the conclusion that shear stress is present in the entire outflow system and that NO and endothelin are major aqueous outflow regulatory factors [5]. The evidence and reasoning follow. In the human outflow system, rapid pulsatile flow into SC, the CCs, intrascleral channels, and the aqueous veins was directly observable, providing reality-based evidence of its presence (Section 2.2). Cyclic pulsatile aqueous flow from the TM into SIV progressed into SC. Pulsatile aqueous ejected into SC. Pulsatility was maintained as aqueous entered CCs and intrascleral channels. The speed of the cyclic pulsatile outflow system motion was synchronous with the cardiac pulse.

The cardiac pulse induces shear stress signals in the endothelium of vessel walls throughout the vascular system. Aqueous outflow system pathways have a comparable vascular endothelium and experience the same pulse speed. Outflow system structures can be expected to use identical mechanisms [29]. Initial evidence in human subjects led to the conclusion that shear stress-dependent nitric oxide signaling must be present in the outflow system in humans. Laboratory studies began seven years later with extensive follow-up studies validating the initial clinical evidence and conclusions, including evidence of effects on distal outflow [56,57,58,59,60,61,62,63,64,65].

## 8. Ciliary Body: Central Role in the Regulation of Aqueous Outflow

### 8.1. The Role of Ciliary Muscles in Preventing SC Collapse and TM Stiffness/Elastance

Ciliary body properties are central to IOP control. The ciliary body and the TM are anatomically and functionally inseparable units. The ciliary body regulates TM distention into SC by continually maintaining tension, preventing persistent SC collapse. Ciliary body tension moves the TM toward the AC then the SC lumen size increases, the spaces between TM lamellae enlarge, and the TM elastance/stiffness increases [8,10,11,41,41,66,67,68,69,70,71,72,73,74].

The tensional stress from SC enlargement increases SIV tension on CC hinged flaps, increasing CC and CDSP lumen dimensions. The improved vector forces enlarge outflow pathways, reducing outflow pathway resistance and improving responsiveness to IOP setpoint deviations as well as oscillatory and pulse transients. Increasing ciliary body tension favors outflow pathway enlargement, and stroke volume increases. Enhancing the dual pump/conduit function can thus reduce IOP.

### 8.2. Ciliary Body Attachments and Geometric Relations That Permit Regulation

The scleral spur is an anterior extension of the sclera that encompasses the posterior portion of SC, resulting in a mobile hinged region. The vector forces associated with scleral spur movement cause it to move inward and posteriorly with ciliary muscle tension. Thus, the ciliary muscle induces stress on the TM lamellae through direct ciliary muscle connections and scleral spur connections. The tension causes rotation of the TM inward, enlarging the SC area. The stress-induced tension also increases interspaces between trabecular lamellae and causes elongation. The ciliary muscle’s geometric position and contractile state determine the TM lamellae’s anatomic position and mechanical properties essential to flow regulation (Figure 2). The importance is illustrated by evidence that clinical disinsertion of the ciliary muscle results in severe angle recession glaucoma [41,75]. For example, see (Video 7) showing the effects of ciliary body tension on TM.

Phenomenological ground truth is easily determined using direct observation. Ciliary muscle tension results in the TM moving away from the external wall of SC and toward the AC. Lamellae move posteriorly, but their attachment to Schwalbe’s line is maintained, resulting in TM lamellae elongation. The elongation increases tensional stress and increases the stored potential energy (Figure 2). Evidence of the lamellae storage of potential energy is evident when ciliary muscle tension is released. The TM lamellae immediately shorten, and the TM complex moves toward the outer wall of SC.

### 8.3. TM Elastance/Stiffness Curve of the TM Controlled by the Ciliary Muscle

Stiffness/elastance of biological tissues is not a static property but is instead tightly linked to instantaneous tissue loading forces. As ciliary muscle tension increases, the TM lamellae move up the elastance curve, reflecting increased stiffness [48]. An elastance curve defines the tissue’s ability to distend and recoil, and in the case of the TM, it involves measuring stiffness under varying loads. For homeostasis, a narrow range of elastance/stiffness must be maintained.

If the TM lamellae are abnormally compliant or stiff, they cannot move appropriately in response to IOP changes. The ciliary muscle is a primary determinant of TM elastance. For example, see the (Video 7) showing TM-ciliary muscle elastance. Optimized TM elastance governs the degree of pressure-dependent TM distention into SC and responses to oscillatory forces such as eye movement/blinking-related transients and associated pulsatile behavior [48].

### 8.4. Opposing Forces of IOP and Ciliary Muscle Tension Control TM Position

Two opposing forces govern the TM’s position in the 3D space between the AC and SC. The compressive forces are (1) IOP, (2) superimposed transient pulses, and (3) oscillatory forces. These forces impinge on SC endothelium, which deforms in response and moves toward the SC lumen. Opposing forces involving tension are (1) the TM lamellae elastance as a tethering force limiting SC endothelial distention, (2) the anterior–posterior geometric position of the ciliary muscle, and (3) the contractile properties of the ciliary muscle. The combined tensile forces continually oppose the compressive forces, providing a homeostatic equilibrium position of the TM.

The TM may be likened to a stringed instrument constantly adjusting its properties to remain in tune. When optimally tuned, its position in 3D space provides an ideal relationship between the inner and outer walls of the SC. At the same time, the optimized elastance permits the TM to dance to the tune of the ocular pulse. Accurate tonal responses to the IOP, IOP transients, and oscillations are necessarily lost with too little or too much tension.

The seamless anatomic connections between TM lamellae and ciliary muscle ensure that vector forces act synchronously. This inextricable linkage requires synchronous dual regulation to maintain optimized SC dimensions, TM elastance, and IOP control. The relationships and elastance/stiffness requirements are necessary to maintain a highly regulated ciliary muscle contractile state. Age-related changes in position and contractile state are thought to alter the ciliary muscle’s ability to participate in the control of IOP [74,76,77].

### 8.5. Ciliary Muscle Control of Distal Pathway Resistance

When ciliary muscle tension increases, SC enlarges, and SIV elongation increases. The SIV elongation transmits increased stress from the TM to septa that function as hinged flaps at CC entrances. The integrated structural CB-TM-SIV-SOV relationships allow the ciliary muscle to alter CC entrance dimensions (Figure 2).

The crystalline lens attachment to the ciliary muscle causes stresses that pull the ciliary muscle anteriorly with progressing age [74]. The anterior movement alters vector forces on the TM and scleral spur, causing them to move anteriorly and outward. The TM moves further into SC, reducing the TM lamellae tensile loading forces. Cataract surgery eliminates stresses pulling the ciliary muscle anteriorly. The new vector forces favor posterior and interior rotation of the scleral spur and ciliary body tendons [11]. These vector forces tend to pull the entire TM away from the external wall of SC, open intertrabecular spaces, and increase the tension or load on the trabecular lamellae (Figure 2).

## 9. Resistance: Physiology and Glaucoma Pathology

### 9.1. Little Resistance Is within the TM: Resistance Results from SC Collapse

The classic physiology studies of Grant and colleagues reveal that there is little resistance within the TM itself. Instead, their studies indicate that resistance results from TM motion that leads to SC wall closure and herniation into the entrances of CCs [8,9,10,11,36,67,68]. Grant’s 1958–1963 studies [13,14] in ex vivo eyes used pressures far outside the normal range. The 25 mm Hg IOP represented the equivalent of an in vivo 33 mm Hg transtrabecular pressure gradient because of the absent 8 mm episcleral venous pressure. The contractile tone of the ciliary muscle was absent, and there was no histologic confirmation of the extent of tissue removal. Grant’s group acknowledged and corrected these severe limitations in the later studies [8,9,10,11,36,67,68,78].

Unfortunately, the earlier work provided a simple, easily modeled TM doctrine, which was later demonstrated to be erroneous but entrenched in textbooks by that time. The ideology remains deeply embedded in the currently used investigative framework and permeates every aspect of research efforts. The typically declarative textbook and research reviews characterize control of outflow resistance as being isolated to a narrow region of the TM’s outer wall. The passive model is not presented as provisional and needing further inquiry but as an issue that has been effectively resolved.

The selective absence of citations to the crucial literature does not permit readers themselves to grasp and process the evidence required to assess uncertainty in the declarative statements. Of greater concern, the declarative approach implies an area of settled science. The approach reduces the likelihood of early-stage investigators and busy clinicians challenging, exploring more broadly, or engaging in deeper inquiry, leading to treatment breakthroughs benefiting patients.

### 9.2. Isolation of Resistance to the TM: A Premise Inconsistent with the Best Evidence

Later studies by Grant’s group refuted the premise that his widely cited earlier techniques can eliminate or even identify resistance limited to the TM. Complete removal of the external wall of SC, called sinusotomy, also removed 75% of the resistance. Many attachments were present between the TM and the external wall of SC, preventing isolation of tissue removal to either wall of the canal [10]. A repeat of the earlier studies, now followed by light and SEM examination, demonstrated that the removal technique damaged the TM-SC external wall connections that were torn and pulled away from the external wall of SC [10,11]. The numerous connections meant that there was no way to use the technique to truly separate TM and distal resistance.

Direct observation demonstrated that increasing IOP caused the TM to distend far into the scleral tunnel after removing the external wall of SC. The distension provided physical, directly observable evidence that the TM is mobile, and its distension can occlude SC [8]. A series of ingenious studies, illustrated in Figure 5, verified that resistance increases resulted from TM motion that caused SC collapse; of critical importance, the studies showed that ciliary body tension prevented SC collapse responsible for the increased resistance [8,9,10,11].

### 9.3. Trabecular Meshwork Stiffness Abnormalities as a Factor in Glaucoma

Ex vivo studies using multiple approaches have identified abnormal TM tissue biomechanics in glaucoma patients. One study used the elastic modulus with finite element modeling and found stiffness was modestly higher in glaucoma eyes. The elastic modulus is the ratio of the force exerted upon a substance to the resultant deformation.

Another parameter used to assess TM motion is the time-dependent shear modulus, a measure of ocular rigidity that assesses the outflow tissue’s viscoelastic properties. The shear modulus is the ratio of shear stress to shear strain. Stress in this setting is the IOP, and strain is the tissue deformation. Recent studies found a highly significant reduction in both the long and short-term shear modulus of the ECM, collagen, and elastin in glaucoma vs. normal tissue. The authors concluded that the outflow tissues in glaucoma eyes are stiffer and less able to respond to dynamic IOP changes [79].

An additional stiffness parameter is load rate stiffening. Blinking, eye movement, and lid squeezing cause abrupt pressure increases that may be modified by load rate stiffening. In the outflow system, baseline tissue stiffness is tightly linked to the static loading force of IOP. The abrupt pressure transients require introducing another recently explored stiffness parameter, load rate-dependent stiffening with a time component.

With rapid movement, tissues stiffen more rapidly, reducing the overall tissue deformation. The stress of the same rise in IOP results in less deformation or strain compared with static conditions. The findings suggest that rate-dependent stiffening may protectively dampen tissue deformation from abrupt pressure transients in patients with normal tissue biomechanics [79].

Another method to assess both TM and CC tissue stiffness is real-time measurements of outflow pathway motion using high-resolution OCT. Evaluation of the complex motion curves using MANOVA demonstrated a significant reduction in movement of both the TM and tissues surrounding CCs in eyes with glaucoma [12].

## 10. Crucial Evidence: IOP, TM Removal, and Ciliary Muscle Tension: Impact on Outflow Facility

Figure 5A illustrates how crystalline lens backward movement dilates Schlemm’s canal (SC) and reduces resistance [8,9,11,68]. The upper panel shows no lens depression. The trabecular meshwork (TM) is in extensive apposition to the external wall (EW) of SC, causing the closure of the SC lumen (arrow). In the lower panel, with depression of the crystalline lens, the ciliary body (CB) and scleral spur (SS) rotate posteriorly, pulling the TM attachments away from the external wall of SC. The TM distends, and the SC lumen is large. The black arrow demonstrates the SC inlet valve extending from the TM to a hinged flap at a collector channel entrance. 

Figure 5B shows that the corneal perfusion fitting contains a lens-depression device. The CB and SS move backward with lens depression, resulting in the opening of SC. As the lens moves backward, causing CB tension, resistance falls by over 50% (Figure 5C(1)). There is no iridectomy. Anterior chamber perfusion forces the lens backward, resulting in a reverse pupillary block phenomenon. The backward movement increases zonular tension that transmits to the ciliary body, scleral spur (SS), and TM tendons. The tension and vector forces cause the scleral spur and TM to move posteriorly and inward. In Figure 5C(2), with an iridectomy, pressure gradients equalize between the anterior and posterior chamber, eliminating posterior lens movement or ciliary body tension. 

Figure 5D shows the outflow facility experimentally controlled at a series of steady-state intraocular pressures. The blue curve is the outflow facility with no ciliary body tension, as shown in the Figure 5C(2) protocol. The orange curve is the outflow facility with ciliary body tension resulting from the protocol, as shown in Figure 5C(1). Pressures in the abscissa need to be adjusted upward by 8 mm Hg to reflect transtrabecular in vivo pressure gradients because of the lack of episcleral venous pressure in the ex vivo setting. Ellingsen and Grant determined the reduction in outflow resistance from TM removal at each pressure, as noted in the blue curve shown in Figure 5C(2). As indicated by the boxed data, an effective IOP of 13, 18, and 33 mm Hg, trabeculotomy reduces resistance (R) by 14%, 27%, and 75%, respectively. The upward-pointing blue arrow and asterisk indicate the conditions of Grant’s initial 1958 and 1963 studies. With simulated ciliary muscle tension as in condition C2, the outflow facility is initially higher than under condition C1. The facility of outflow remains high despite increasing IOP. The authors concluded that the apposition of the TM to the SC external wall caused increased resistance with increased pressure. They also concluded that ciliary muscle tension prevents SC wall apposition and associated reduction in outflow facility.

## 11. Identifying Outflow Structure Damage When Planning Surgical Intervention

### 11.1. Clinical Angiography: Office-Based Hemoglobin Video Editing

Pulsatile flow from the aqueous to the episcleral veins is readily visible using a slit lamp. Investigative studies with the technique provide clinicians with remarkable insights into physiologic outflow mechanisms [3,28]. Because it is very time-consuming, the slit lamp approach is of limited value in clinical settings.

Hemoglobin video imaging is a newly developed technique adapted for use at the slit lamp in a clinical environment. The technique provides noninvasive, real-time, high-resolution images. The approach can differentiate normal from glaucoma patients by quantifying the flow rate [80,81,82,83]. The hemoglobin absorption spectrum increases the contrast of red cells, improving the distinction between episcleral venous blood and aqueous. By studying flow patterns in the clinic, it may be possible to identify optimal MIGS placement locations before going to the OR.

### 11.2. Functional Outflow System Abnormality Assessment Using Phase-OCT

We lack knowledge of the crucially important IOP profile in individual patients. IOP measurements are inherently suboptimal. Office IOP readings occur at random times and are of brief duration, involving about 16 s of 31 million seconds in a year. Diurnal variation is not captured. In addition, there is unknown patient compliance, and the range of ocular transients is unknown.

In contrast, the outflow system’s underlying biomechanical properties that permit it to maintain IOP homeostasis are intrinsic tissue properties. These biomechanical properties determine the TM’s ability to distend and recoil. The PhS-OCT system enables identifying movement with a sensitivity of 20 nanometers with ~ a 1000 nanometer dynamic range. Tracking and quantitative measurements of real-time pulse-dependent TM motion are now possible in patients with the recently developed Phase-OCT system. The system identifies differences between glaucoma and normal patients, performing better than IOP or tonography measurements [84]. The Phase-OCT technique also identifies diurnal pressure variability not captured in random office measurements.

### 11.3. Introduction of a Fluid Wave to Assess MIGS Placement and Surgical Success

During MIGS surgery, infusing a fluid bolus into the AC displaces blood in the episcleral veins, causing the clear fluid to create a pulsatile episcleral vein bolus and regional blanching. The pulsatile wave and blanching demonstrate patency of structural pathways from the AC to the aqueous and episcleral veins. These clinical findings are predictive of surgical success [85]. Other operating approaches introduce dyes into the AC or SC. These studies provide evidence of the pulsatile behavior of aqueous flow in both NH primates and humans. The approach shows promise in identifying the proper location for MIGS placement [86,87,88].

### 11.4. MIGS Reduction in IOP Is Not by Physiologic Mechanisms

Canal-based MIGS procedures avoid the risks of trabeculectomy surgery. The procedures represent a valuable interim contribution to glaucoma patient care while awaiting a more definitive problem resolution based on an improved understanding of outflow system physiology. Current MIGS procedures cannot restore physiologic pressure control mechanisms because they disrupt structures within SC involved in IOP control [4,36] (Figure 7). Nonetheless, the importance of MIGS is not diminished by their lack of targeting physiologic IOP control mechanisms.

Some SC-directed MIGS procedures remove much of the TM, and others cannulate the canal. The currently available cannulas are too large to fit within the confines of SC. These cannulas disrupt SC inner wall endothelium, simultaneously tearing the anterior and posterior walls at their attachment sites. A cannula passage also necessarily disrupts SIVs and attachments to SOV [6,10,11,15].

Well-defined structural features distal to the TM include SIV, septa and their hinged flaps at CCs, and the circumferentially oriented collapsible chamber surrounding SC. Such structural pathways are involved in regulating resistance distal to the TM. MIGS procedures ablate or disrupt these pressure-regulating structures at the TM and SC levels. However, TM flaps may be left behind, and the septa that maintain CDSP dimensions are not reliably removed. Even after circumferential trabeculotomy and canaloplasty, short-term IOP fluctuation may be high [89].

A recent study examined the effects of disrupting SIV linkage to the hinged flaps at SOV by injecting a fluid bolus into SC. The SOV channel dimensions were decreased after SIV connections to the TM were disrupted. Outflow tissue biomechanics were substantially altered, as illustrated by the quantitative changes in TM elastance [48]. Altered TM elastance necessitates alterations in physiologic pressure and pulse-dependent responses involved in IOP control.

## 12. Recovery of Normal Function—Requirements

### 12.1. Problem Identification as the First Step

In medicine, the nature of the problem must be identified before a rational solution can be implemented. Identification of the tissues causing IOP abnormality in glaucoma remains elusive. However, studies consistently show that alteration in stiffness/elastance of the TM-ciliary muscle complex is a primary factor

We reason that outflow tissues stiffen like other vascular tissues, such as the aorta, subjected to constant oscillatory forces. Age-related tissue stiffening results from elastin fragmentation and replacement with collagen, which is 100 times less distensible than elastin. An illustration is age-related elastin replacement with collagen in the systemic vasculature, leading to the vessel wall stiffening that we call arteriolosclerosis. Aortic enlargement occurs due to vessel wall stiffening, leading to the inability to recoil. Pulsatile pressure subjects the aorta to continuous oscillations. The stiffening leads to aortic expansion and a 50% enlargement in diameter between ages 40 and 70 [29].

Elastin fragmentation and replacement with collagen is a systemwide age-related phenomenon likely to be shared by the TM lamellae. The TM lamellae are part of a vessel wall that we call SC. The lamellae experience constant pressure forces and oscillations like other vessel walls that result in progressive distention with aging. Vessel wall distention in the case of the TM results in outward movement into SC, narrowing its lumen. Such changes may result in persistent SC closure in glaucoma patients. When SC wall apposition persists, the TM loses its equipoise in 3D space. If the walls of SC become persistently appositional, the TM’s ability to sense or respond to pressure changes is lost (Figure 2). In glaucoma, TM motion (Section 2.4) and pulsatile flow (Section 3.1) slow and then stop.

### 12.2. Problem Resolution as the Second Step

A reasonable goal for addressing the IOP problem in glaucoma is the restoration of a low, minimally fluctuating IOP. Current evidence indicates that optimized tissue elastance/stiffness restoration should restore homeostasis to normal. Techniques for identifying abnormal elastance and restoring it to normal are evolving [16,48,80,81,82,83].

### 12.3. Restoration of Normal Outflow System Elastance and IOP in Glaucoma

A practical illustration of restoration of elastance in glaucoma patients is the response to miotics. Prestress induced by IOP causes the entire outflow system to function as a tensionally unified complex. (Section 4.4). Ciliary muscle position and contractile properties control tensional loading forces, ensuring uniform distribution throughout the entire system from the TM to the level of the septa at CC entrances (Video 7) [4]. Ciliary muscle tension increases the loading force on the TM lamellae, causing them to move up the elastance curve, resulting in greater stiffness. At the same time, the TM moves toward the AC, enlarging SC [11,48,67,68].

Directly visible evidence of increased pulsatile flow in the aqueous veins can be correlated with known miotic-induced structural responses within the outflow system. Following the miotic-induced increase in pulsatile flow, IOP falls to a new temporary homeostatic setpoint. The increase in pulsatile flow followed by a reduction in IOP is compelling evidence of an elastance-related functional abnormality in glaucoma [1,3,26].

The miotic response provides robust evidence of the functional abnormality in glaucoma. It also furnishes clues to restoring normal outflow on a more permanent basis. Three techniques target the tension-dependent ciliary muscle or TM lamellae by introducing heat. ALT, SLT, and micropulse lasers are each designed to induce heat. Increased temperatures induce collagen shrinkage that can target the tension-dependent TM lamellae or ciliary muscle [90]. Cataract surgery and scleral expansion alter ciliary muscle tension and can lower IOP [91].

### 12.4. Cataract Surgery

One cataract-only control arm of a prospective randomized trial found a mean IOP reduction of 8.5 ± 4.3 mm Hg at 13 months. The OHTS study provided a rigorously controlled and characterized assessment of the effect of cataract surgery and found that IOP was reduced by greater than 4.0 mmHg (16%) [74,91,92].

A recently proposed explanation for the IOP reduction following cataract surgery is that it restores the aqueous outflow pump’s sensory and motor properties that regulate IOP. Cataract surgery causes the AC to deepen, and the position of the residual lens capsule is posterior to SC. The new vector forces rotate the TM and scleral spur attachments backward in response to ciliary muscle tension, increasing tension in the trabecular tendons [74]. The new vector forces alter the effect of ciliary muscle tension. The TM interspaces enlarge, and SC’s lumen volume increases, as shown in a recent OCT study in patients [93]. The restored configuration is more like that in youth, as revealed using high-resolution MRI [74,91]. The advantage of cataract removal is that no procedure-related outflow system structural damage occurs.

### 12.5. ALT, SLT, and Micropulse Laser as Elastance Improving Procedures

The TM lamellae–ciliary muscle complex is a tensionally integrated prestressed system. The trabecular lamellae are collagenous, and the ciliary muscle contains extensive fascial collagen components. Heating of collagen induces shrinkage. Heating that causes collagen shrinkage anywhere along the system necessarily transmits added tension to the entire complex. The elastin–collagen transition that diminishes elastance with age reduces the ability to respond to pressure gradients. Collagen shrinkage that induces tissue tightening may restore more normal IOP responses. Argon laser trabeculoplasty (ALT) causes heat-induced TM lamellar collagen shrinkage [94]. Selective laser trabeculoplasty (SLT) also heats outflow system tissues.

Pigmented cell death with cytokine release is a proposed mechanism for SLT’s ability to reduce IOP. When heat is sufficient to kill cells, collagen shrinkage can also be expected. Histologic studies would not easily detect the small changes necessary to alter elastance [95]. Although subtle tissue shrinkage cannot be identified using histology, shrinkage can be identified using direct real-time same-sample imaging as achieved with a micropulse laser.

Cytokine release after an injury occurs rapidly and is of relatively brief duration. However, ALT and SLT effects on IOP often persist for many months to several years. Heat-induced tissue shrinkage during laser genioplasty is routinely observed and has a similar long-lasting effect. The relatively long duration of the SLT effect on IOP can be more easily explained by its ability to induce collagen shrinkage than the short-term effects of cytokine release [96].

The transscleral micropulse laser procedure causes ciliary muscle shrinkage and a change in TM configuration that is remarkably similar to the effects of miotics. It is essential to differentiate the micropulse technique from traditional procedures that use high energy to destroy the secretory ciliary endothelium. The laser penetration is too limited with the micropulse technique, so damage to the ciliary epithelium does not occur because the laser does not penetrate to its depth [97,98].

The micropulse procedure may prove attractive as a noninvasive means for restoring normal outflow function in glaucoma. Technique improvements can improve the success rate and reproducibility. Targets for parameter improvements include beam focus, wavelength, duty cycle, energy, and the development of a highly reproducible laser delivery system.

## 13. Limitations

Our report’s crucial limitation is its reliance on studies from experimental platforms developed in our laboratories. Independent confirmation of the presence of the distal circumferential channels able to act as valves is lacking. The new awareness of the Ramirez group’s work provides confirmatory evidence. It establishes the circumferential “outer collectors” as normally occurring structures in humans with anatomic and functional significance [50].

Another of this report’s limitations was our use of controlled reverse perfusion of SC to study the dynamics of transtrabecular pressure gradients that had not been validated by an independent group. However, recently, another group successfully used the SC perfusion technique to study outflow system dynamics [79]. Their study demonstrated the platform’s ability to explore the outflow pathway, finding a rate-dependent loading boundary with large fluctuations consistent with our findings.

Although anatomy, structural relationships, and motion are becoming clearer, much more work is needed to refine the tissue motion details. Our (Video 9) animation depicts one possible sequence of pulsatile flow, but it is only one of many scenarios and remains a wide-open question that is not easily resolvable with current technologies. The need to continually update the model illustrates our conceptual framework’s evolving and provisional nature. We accept the need to maintain a degree of uncertainty and expect the model to undergo substantial modifications as new imaging technologies provide a more detailed picture of the outflow system’s dynamic behavior.

## 14. Summary

The aqueous outflow system is a vascular circulatory loop. We present evidence leading to the conclusion that physiologic mechanisms in the rest of the vasculature also control aqueous outflow and intraocular pressure. In support of our conclusions, we consider readily verifiable reality-based knowledge from direct observation of pulsatile flow and its abnormalities in humans.

We describe pulsatile tissue motion, chambers, and valves that act synchronously to control the stroke volume of aqueous discharge into the venous system. We proposed that the outflow tissues function as a pump–conduit system similar to lymphatics. Our work introduces physiologic mechanisms involving pulse-dependent tissue and cellular motion as a sensory stimulus providing constant feedback to maintain a genomically determined intraocular pressure setpoint.

Our development of the pump conduit model also introduced the concept of pulse-dependent shear stress to regulate outflow pathway diameters through NO/endothelin pathways. We also introduced the role of the glycocalyx as a shear stress sensor, a barrier to prevent fluid passage across SC endothelium, and a means of ensuring load-bearing properties of the endothelium that enable its pressure-dependent deformation

We further introduced evidence of cellular attachments of the SC endothelium to the TM lamellae. The attachments permit us to introduce principles from vascular physiology, including pressure-dependent tensional integration and prestress central to mechanotransduction mechanisms that maintain homeostatic pressures throughout the vascular system. The constellation of findings leads us to conclude that the proposed behavior provides a rational basis to explain the regulation of aqueous outflow and intraocular pressure.

## Figures and Tables

**Table 1:** Videos and descriptions. Click either item number or thumbnails for links to videos. (SC) Schlemm’s canal, (IOP) intraocular pressure, (SIVs) Schlemm’s canal inlet valves, (AC) anterior chamber, (CC) collector channel, (HR-OCT) high-resolution OCT, (ISCC) intrascleral collector channel, (SOV) SC outlet valve.

#	What the Videos Demonstrate: Pulsatile Flow and Outflow Tissue Motion	Links
1	• Aqueous exiting SC enters aqueous veins in pulsatile waves. • Propagating aqueous waves cyclically enter episcleral veins.	** 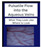 **
2	• Increase in IOP increases pulse stroke volume, discharging more aqueous. • Increases aqueous discharge, then reduces IOP to setpoint. A feedback loop.	** 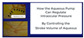 **
3	• SC inlet valve (SIV) lumen is a direct AC to CC aqueous conduit. • Perfused tracers are in the TM, SIV, and CC.	** 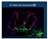 **
4	• Synchronous pressure-dependent TM and distal outflow channel motion. • Fresh tissue and HR-OCT. HR-OCT is at 05:27 in the video.	** 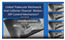 **
5	• SC inlet valve relationships and elasticity shown in an operating room @ 00:53. • Pulsatile aqueous flow from the anterior chamber into SC @ 02:19.	** 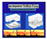 **
6	• Pulse waves directed at a limbal segment cause TM distention and recoil. • The TM rapidly distends into SC storing elastic energy. Recoil follows.	** 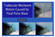 **
7	• Ciliary muscle tension in the radial limbal section opens SC and CC. • Tension causes TM lamellae elongation, rotation toward AC, and then recoil.	** 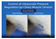 **
8	• TM motion is synchronous with SC, CC, and ISCC (SOV) volume changes. • In a glaucoma eye, TM motion slowed and was limited by SC wall adhesions	** 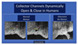 **
9	• TM position is tightly linked to IOP changes with baroreceptor-like behavior. • TM movement opens distal valves to control aqueous discharge.	** 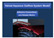 **

## Abbreviations

trabecular meshwork (TM), Schlemm’s canal (SC), SC inlet valve (SIV), SC outlet valve (SOV), optical coherence tomography (OCT), phase OCT (PhS-OCT), spectral domain OCT (SD-OCT), scanning electron microscopy (SEM), minimally invasive glaucoma surgery (MIGS), circumferential vascular channel (CVC), circumferential deep scleral plexus (CDSP), episcleral vein pressure (EVP), aqueous vein pressure (AVP).
